# Motor skill learning and reward consumption differentially affect VTA activation

**DOI:** 10.1038/s41598-017-18716-w

**Published:** 2018-01-12

**Authors:** Susan Leemburg, Tara Canonica, Andreas Luft

**Affiliations:** 1 Division of Vascular Neurology and Rehabilitation, Department of Neurology, University Hospital Zurich, Zurich, Switzerland; 2 Cereneo Center for Neurology and Rehabilitation, Vitznau, Switzerland; 3 College of Biomedical and Life Sciences, School of Psychology, Cardiff University, Cardiff, United Kingdom

## Abstract

Dopamine release from the ventral tegmental area (VTA) terminals in the primary motor cortex (M1) enables motor skill acquisition. Here, we test the hypothesis that dopaminergic VTA neurons projecting to M1 are activated when rewards are obtained during motor skill acquisition, but not during task execution at plateau performance, or by rewards obtained without performing skilled movements. Rats were trained to perform a skilled reaching task for 3 days (acquisition) or 7 days (plateau). In combination with retrograde labelling of VTA-to-M1 projection neurons, double immunofluorescence for c-fos and tyrosine hydroxylase (TH) was used to assess activation of dopaminergic and non-dopaminergic VTA neurons. Dopaminergic VTA-to-M1 projection neurons were indeed activated during successful motor skill acquisition, but not when rats failed to learn or had reached plateau performance, nor by food rewards alone. By contrast, dopaminergic VTA neurons that did not project to M1 were activated by both skilled reaching and food rewards. Non-dopaminergic neurons were found to be activated by motor task performance at plateau, but not during skill acquisition. These results indicate that distinct populations of VTA neurons are activated by motor skill acquisition and task performance. Moreover, this activation is not merely related to consumption of food rewards.

## Introduction

Dopamine release from the ventral tegmental area (VTA) to the nucleus accumbens and the frontal cortex plays an important role in reward processing and supports a variety of reinforcement learning processes^1,2^. In the initial stages of learning, VTA activation reflects reward reception (e.g. food), while conditioned stimuli (e.g. light or sound cues) more effectively activate VTA in later learning stages^3,4^. Inhibition of VTA activity prevents task acquisition^1,3,5,6^, suggesting an essential role for VTA dopamine release in a variety of learning tasks.

Motor skill acquisition, but not execution of an already learned skill, depends on dopamine release from VTA projections to the primary motor cortex (M1)^7,8^. Like other VTA efferents, those to M1 are highly specific and send very few collaterals to other brain regions^9,10^. VTA neurons projecting to M1 do not simultaneously project to nucleus accumbens (NAc) or prefrontal cortex (PFC)^11^. Beyond being anatomically distinct, it is unknown whether VTA-to-M1 projections are also functionally distinct from other projections.

Here, we test the hypothesis that activation of VTA-to-M1 dopaminergic neurons is specifically induced by motor skill acquisition, but not by task performance at plateau after learning has ended, or by reward consumption that does not require skilled movements. After retrograde labelling of the VTA-M1 projection, rats were trained to perform a skilled reaching task. Then, double immunofluorescence for c-fos and TH was used to assess activity of dopaminergic and non-dopaminergic VTA neurons after successful task acquisition, unsuccessful task acquisition and plateau performance. Each experiment included a matched control group of rats that received a food reward at the same time as the paired skilled reaching rat, as well as an untrained, unrewarded control group.

## Results

### Tracer injection and immunofluorescence

**Figure 1:**
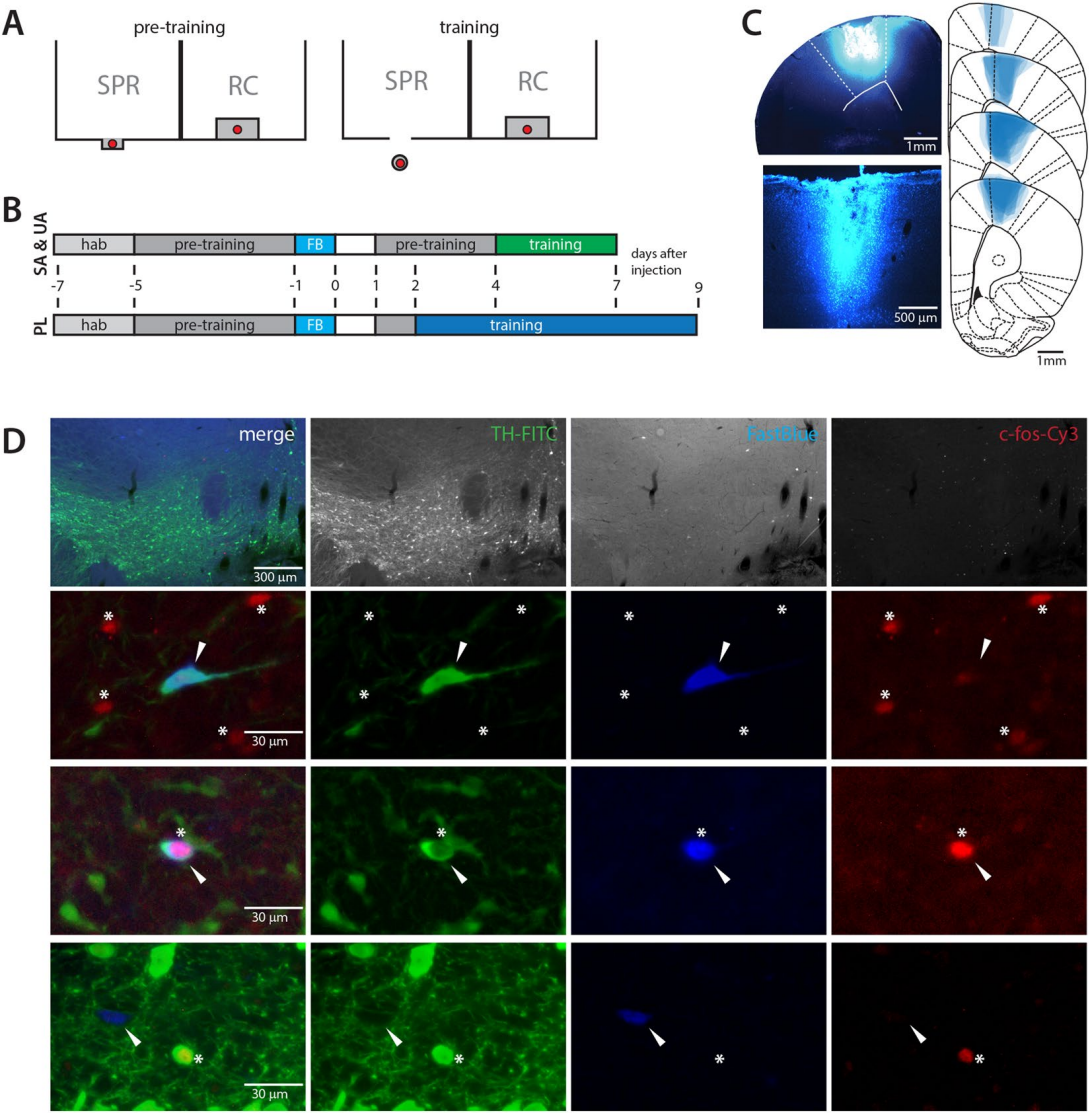
Overview of experimental setup and typical histological results. (**A**) Schematic drawing showing the front of the training apparatus and placement of food pellets (red) during pre-training and single pellet reaching (SPR) training for SPR rats (left part of the cage) and RC rats (right part of the cage). The pellet is placed within tongue-reach for SPR rats during pre-training and on a pedestal, that can only be reached by grabbing during single pellet reaching training. For RC rats, pellets are delivered to the inside of the cage whenever the SPR rat consumes one in both the pre-training and training phase. (**B**) Schematic of experimental setup for successful acquisition (SA), unsuccessful acquisition (UA) and plateau (PL) experiments. Numbers indicate time after FastBlue injection (FB). hab = habituation, training = single pellet reaching training. (**C**) Distribution of FastBlue in M1 after cortical injection. Representative photomicrographs showing dye injection and needle track in cortex (left) and extend of dye spread in sections 2.7-2 mm rostral of bregma (right). (**D**) Representative examples of VTA neurons positive for tyrosine hydroxylase (TH-FITC, green), FastBlue (FB, blue) and/or c-fos (c-fos-Cy3, red). Top row: 10x magnification, bottom rows: 40x magnification. Arrows indicate FB^+^ cell bodies. Asterisks denote position of c-fos^+^ nuclei.

8-9 days after injection, FastBlue (FB) was detected around the injection sites in deep and superficial cortical layers. Most of the tracer was found within M1 and had not spread to subcortical areas, although a small amount of FastBlue was present in secondary motor cortex directly adjacent to M1 (Fig. 1C). FB^+^ cell bodies were detected in the VTA, some of which expressed tyrosine hydroxylase (TH), c-fos, or both (Fig. 1D).

### Behaviour

**Figure 2:**
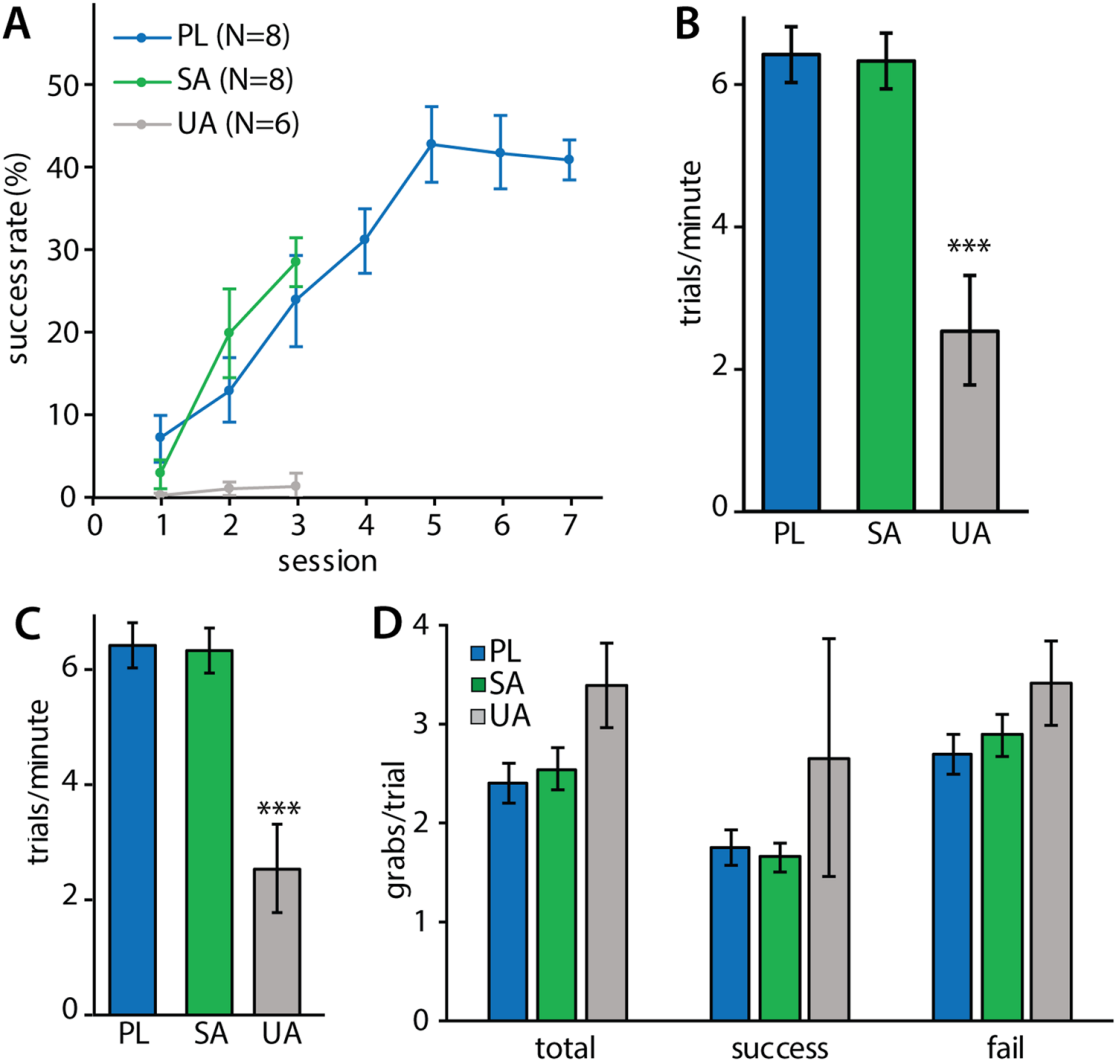
Behaviour of SPR rats during task execution in SA (N = 8), UA (N = 6) and PL (N = 8) experiments. Values are mean ± s.e.m. (**A**) Learning curves showing percentage of successfully obtained pellets per training session. (**B**) Distance walked during the final training session. (***p ≤ 0.001 in Tukey’s HSD test) (**C**) Number of trials executed per minute during the final training session. (***p ≤ 0.001 in Tukey’s HSD test) (**D**) Number of grabbing attempts per trial in the final training session of SA (N = 8), UA (total and fail: N = 5 of 6, success: N = 2 of 6) and PL (N = 8) rats. 3 rats in the UA experiment did not achieve any successful trials.

Rats that were trained to perform the single pellet reaching task (SPR) in the successful acquisition (SA) and plateau (PL) experiments acquired the reaching task at a similar rate. Their reaching performance improved significantly during the first 3 training sessions (two-way repeated measures ANOVA, experiment effect: F(2) = 7.78, p = 0.003; session effect: F(2) = 26.56, p < 0.001; experiment*session interaction: F(4) = 6.50, p < 0.001; Fig. 2A). Rats in the unsuccessful acquisition experiment (UA) did not show any improvement during this period. Rats in the plateau experiment reached plateau pellet reaching performance by session 5. Final success rates in session 3 for acquisition experiments and in session 7 for plateau, differed significantly between all three groups (successful acquisition: 28.8 ± 2.9%; unsuccessful acquisition: 1.6 ± 1.4%, plateau: 41.2 ± 2.4%; one-way ANOVA F(2) = 65.39, p < 0.001; Fig. 2A). Rats in the successful acquisition and plateau experiments had similar levels of overall motor activity: they completed the same number of trials during their final training session and performed the task at a similar speed. Rats that did not successfully learn to reach for food (UA experiment) were not only slower than successful learners (trials per minute, one-way ANOVA F(2) = 17.18, p < 0.001; Fig. 2B), but also completed fewer trials (successful acquisition: 200, unsuccessful acquisition: 105 ± 32.7, plateau: 200). As a result, unsuccessful learners also walked a shorter distance during their final training session than animals in the successful acquisition and plateau experiments (one-way ANOVA F(2) = 11.50, p = 0.001; Fig. 2C). Forelimb specific motor activity was analysed by counting grabbing attempts, defined as an extension of the paw towards the pellet followed by complete or partial retraction. The number of grabbing attempts per trial during the final training session was not significantly different in the three experiments (total: one-way ANOVA F(2) = 3.51, p = 0.052, successful trials: one-way ANOVA F(2) = 1.60, p = 0.23, failed trials: one-way ANOVA F(2) = 1.66, p = 0.22; Fig. 2D). Overall motor activity and forelimb specific activity were not significantly different in rats in the successful acquisition and plateau experiments.

### Effects of successful motor skill acquisition (SA) on dopaminergic VTA neurons

**Figure 3:**
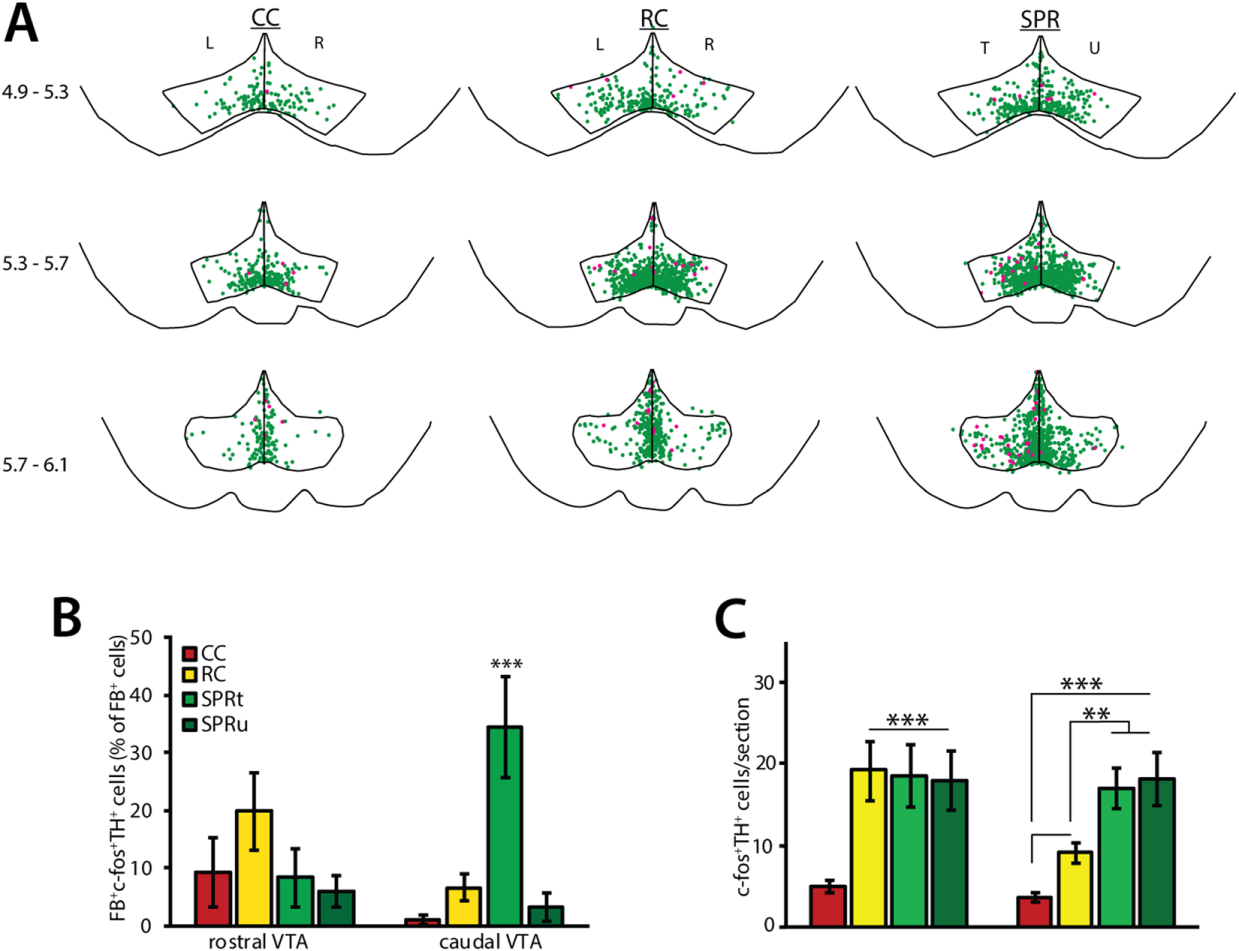
c-fos expression in dopaminergic neurons during successful acquisition (SA, N = 8 per group). SPR = single pellet reaching, RC = reward control, CC = cage control. T = trained, U = untrained, L = left, R = right hemisphere. Asterisks indicate significant differences (*p < 0.05, **p < 0.01, ***p ≤ 0.001 in Tukey’s HSD test), values are mean ± s.e.m. (**A**) Superposition of TH^+^c-fos^+^ (green) and FB^+^ TH^+^c-fos^+^ (pink) neurons in VTA derived from 8 animals (2 sections per position per animal). Numbers indicate position relative to bregma. (**B**) Number of FB^+^ TH^+^c-fos^+^ neurons in rostral and caudal VTA sections. (**C**) Number of TH^+^c-fos^+^ neurons in rostral and caudal VTA sections.

Brain sections of three groups of rats were analysed to assess the effects of motor skill learning and reward consumption on VTA activity. Reaching rats were trained to perform the single pellet reaching task (SPR). Reward control rats received a food reward whenever the reaching rat successfully obtained one, but did not need to use their paws to reach for it (Fig. 1A). This group served mainly as a control for reward consumption. Finally, a group of rats that was not trained and did not receive any food rewards was included to provide a measure of baseline VTA activity. Cage control (CC) and reward control (RC) rats showed equal numbers of immunopositive VTA neurons in left and right hemispheres. Because these animals did not perform unilateral tasks, cell counts for the left and the right hemisphere were averaged. Trained and untrained hemispheres of reaching rats (SPR) were analysed separately. C-fos expression was analysed separately for rostral VTA (4.9–5.5 mm caudal of bregma) and caudal VTA (5.5–6.3 mm caudal of bregma), because these subregions have previously been shown to play differential roles in reward-related signaling^12–20^. C-fos expression was found throughout the VTA in dopaminergic (TH^+^) neurons (Fig. 3A) and non-dopaminergic (TH^−^) neurons (Fig. S1) in all three groups. As described previously, a small number of dopaminergic VTA neurons project directly to M1 and are required for motor skill learning^8^. Indeed, the overall percentage of c-fos^+^ dopaminergic VTA neurons that project to M1 (FB^+^TH^+^c-fos^+^ as percentage of the total number of FB^+^ neurons) was specifically increased in trained hemisphere of rats that successfully learned to reach for food (SPR; trained hemisphere: 24.8 ± 7.2%; untrained hemisphere: 4.6 ± 1.9% of all FB^+^ neurons), compared to rats that received food rewards without reaching for them (reward control, 9.8 ± 2.7%) and rats that did not (cage control, 3.1 ± 1.8%; one-way ANOVA F(3) = 6.29, p = 0.002, Fig. 3A). Activity in the dopaminergic VTA-M1 projection showed a clear rostro-caudal gradient (Fig. 3A): the percentage of FB^+^TH^+^c-fos^+^ neurons was increased in the trained hemisphere of reaching rats in caudal (one-way ANOVA, F(3) = 10.44, p < 0.001, Fig. 3B), but not in rostral VTA (one-way ANOVA, F(3) = 1.24, p = 0.11, Fig. 3B). Reward consumption unrelated to motor task acquisition did not result in increased c-fos expression in FB^+^ TH^+^ VTA neurons in reward controls (Fig. 3B). Thus, hemisphere-specific activation of caudal dopaminergic VTA-M1 projection neurons seems related to successful acquisition of a motor skill, independently of food reward. Activation of the VTA- M1 projection during successful motor skill acquisition was limited to dopaminergic neurons: the overall percentage of c-fos^+^ non-dopaminergic FB^+^ neurons was very low in reaching rats (SPR, trained hemisphere: 7.81 ± 3.61%, untrained hemisphere: 3.78 ± 1.84%), cage control (CC, 6.77 ± 3.15% of FB^+^ neurons), and reward control rats (RC, 3.94 ± 2.46%), and did not differ significantly between groups (FB^+^TH^−^c-fos^+^; one-way ANOVA, F(3) = 0.58, p = 0.22). C-fos expression in dopaminergic VTA neurons that do not project to M1 (FB^−^TH^+^c-fos^+^) was mostly restricted to medial and ventromedial VTA in untrained cage control rats. In rats that consumed food rewards, either by reaching for them (SPR) or not (reward controls), double-labelled neurons were also found in more lateral and dorsal VTA subregions (Fig. 3A). Both successful motor skill acquisition in reaching rats and reward consumption in reward control rats resulted in increased numbers of FB^−^TH^+^c-fos^+^ neurons in rostral VTA (one-way ANOVA, F(3) = 6.17, p = 0.001; Fig. 3C). By contrast, the caudal VTA showed more activation of dopaminergic neurons in reaching rats than in reward control and cage control rats in both the trained and untrained hemisphere (FB^−^TH^+^c-fos^+^, one-way ANOVA, F(3) = 15.75, p < 0.001; Fig. 3C), a smaller increase was also found in reward control rats. Thus, food rewards increase activity of rostral and caudal VTA dopaminergic neurons, irrespective of how the reward is obtained (i.e. by forelimb reaching or by mouth). Successful acquisition of skilled reaching, however, results in additional activity in caudal VTA. While caudal VTA activation was highest in rats that successfully learned to reach for food, the number of FB^−^TH^+^c-fos^+^ cells in the caudal VTA was not significantly correlated to reaching success during session 3 (Pearson’s R = 0.274, n.s., n = 8).

### Effects of motor skill execution at plateau (PL) on dopaminergic VTA neurons

**Figure 4:**
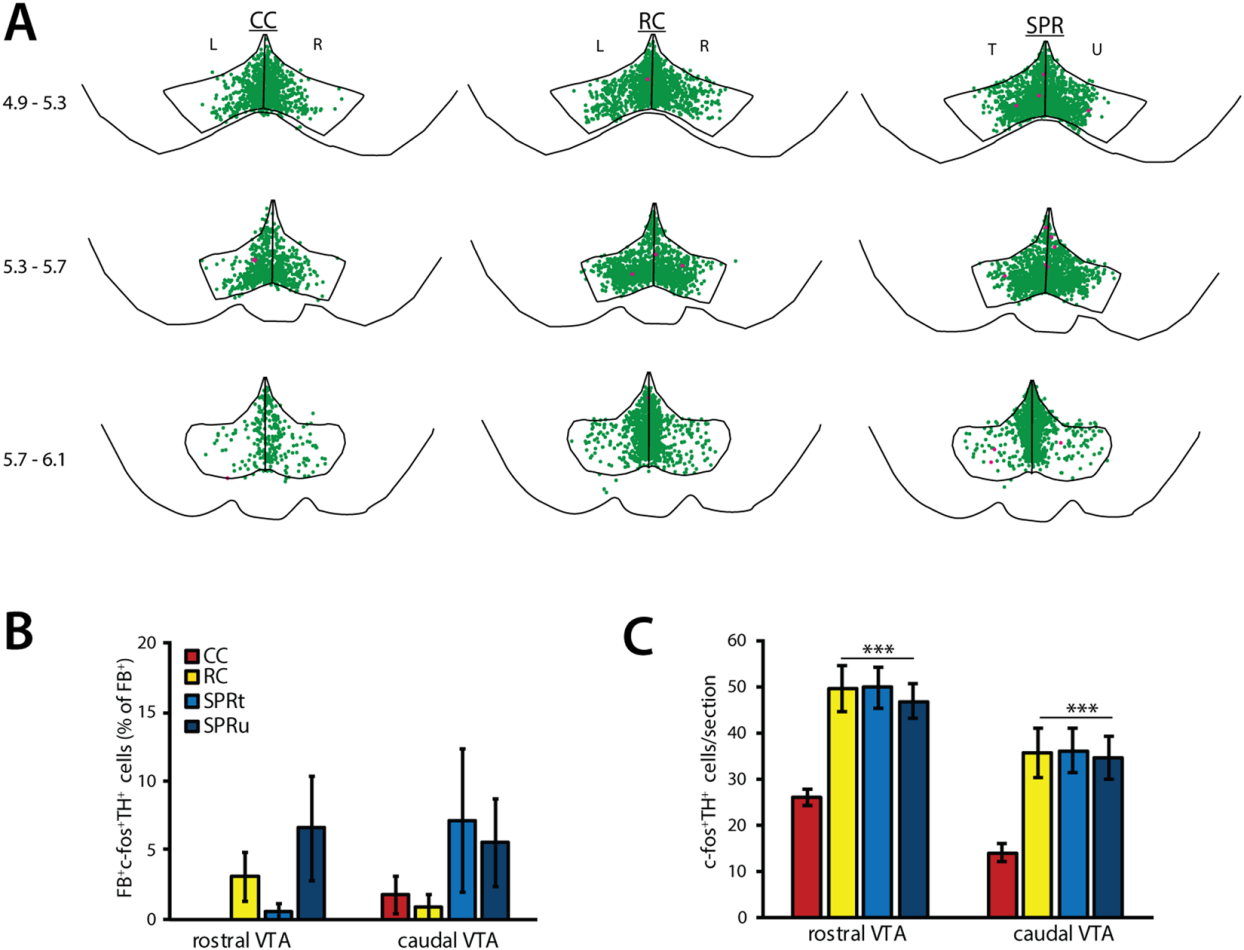
c-fos expression in dopaminergic neurons during plateau task execution (PL, N = 8 per group). SPR = single pellet reaching, RC = reward control, CC = cage control. T = trained, U = untrained, L = left, R = right hemisphere. Values are mean ± s.e.m. (**A**) Superposition of TH^+^c-fos^+^ (green) and FB^+^ TH^+^c-fos^++^ (pink) neurons in VTA derived from 8 animals (2 sections per position per animal). Numbers indicate position relative to bregma. (**B**) Number of FB^+^TH^+^c-fos^+^ neurons in rostral and caudal VTA sections. (**C**) Number of TH^+^c-fos^+^ neurons in rostral and caudal VTA sections. (***p ≤ 0.001 in Tukey’s HSD test).

At plateau, c-fos expression was found throughout the VTA in dopaminergic (TH^+^) neurons (Fig. 4B) and non-dopaminergic (TH^−^) neurons in all three experimental groups. In contrast to acquisition, motor task execution at plateau performance did not result in increased activation of dopaminergic VTA-M1 projection neurons in reaching rats, nor did reward consumption in reward controls (FB^+^TH^−^ c-fos^+^; rostral VTA: one-way ANOVA, F(3) = 2.35, p = 0.094; caudal VTA: one-way ANOVA, F(3) = 1.04, p = 0.39; Fig. 4A,B). C-fos expression in non-dopaminergic VTA-M1 projection neurons (FB^+^TH^−^ c-fos^+^) was likewise low in cage control (CC, 0.19 ± 0.20% of FB^+^ neurons), reward control (RC, 1.67 ± 0.87%) and reaching rats (SPR, trained hemisphere: 2.86 ± 1.80%, untrained hemisphere: 0.91 ± 0.75%) and was unaffected by reward or skilled reaching (one-way ANOVA, F(3) = 1.30, p = 0.29). In contrast to the VTA-M1 projection, c-fos expression in dopaminergic VTA neurons that do not project to M1 (c-fos^+^FB^−^TH^+^) was similarly elevated in both reward control and reaching rats compared to cage control rats in both rostral VTA (one-way ANOVA, F(3) = 9.44, p < 0.001, Fig. 4C) and caudal VTA subregions (one-way ANOVA, F(3) = 6.94, p = 0.001, Fig. 4C). That is, food reward consumption at plateau resulted in similarly increased VTA c-fos expression in rats that reached for food (SPR), as well as rats that did not (RC). Where the number of caudal VTA FB^−^TH^+^c-fos^+^ neurons was higher in reaching rats than in reward controls during successful acquisition, no significant differences were found between these groups at plateau. Moreover, rostro-caudal and medio-lateral distribution of FB^−^TH^+^c-fos^+^ neurons was similar in both groups (Fig. 4A) and did not show the rostro-caudal functional division seen in the successful acquisition experiment. The total number of FB^−^TH^+^c-fos^+^ was not significantly correlated with reaching success rate on training day 7 (SPR; trained hemisphere: Pearson’s R = 0.24, p = 0.57; untrained hemisphere: Pearson’s R = 0.25, p = 0.54, n = 8), or with eaten pellets in reward control rats (Pearson’s R = 0.53, p = 0.16, n = 8).

### Effects of unsuccessful motor skill acquisition (UA) on dopaminergic VTA neurons

**Figure 5:**
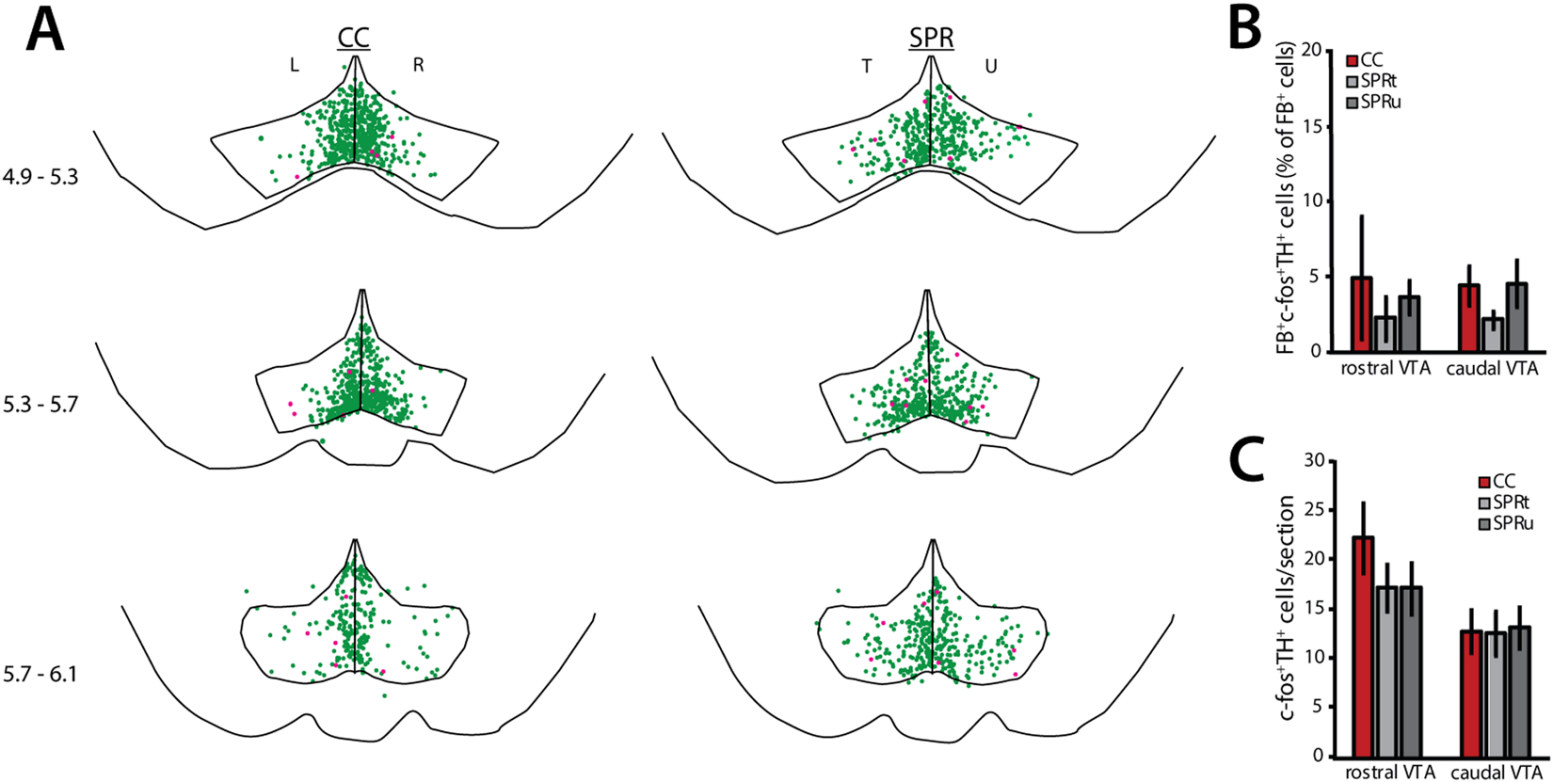
c-fos expression in dopaminergic neurons during unsuccessful acquisition (UA, N = 6 per group). SPR = single pellet reaching, CC = cage control. T = trained, U = untrained, L = left, R = right hemisphere. Values are mean ± s.e.m. (**A**) Superposition of TH^+^c-fos^+^ (green) and FB^+^TH^+^c-fos^+^ (pink) neurons in VTA derived from 6 animals (2 sections per position per animal). Numbers indicate position relative to bregma. (**B**) Number of FB^+^ TH^+^c-fos^+^ neurons in rostral and caudal VTA sections. (**C**) Number of TH^+^c-fos^+^ neurons in rostral and caudal VTA sections.

Like task execution at plateau, unsuccessful motor skill acquisition in rats that were trained to reach for food (SPR) was not associated with increased numbers of c-fos^+^ dopaminergic neurons projecting to M1 (FB^+^TH^+^c-fos^+^; rostral VTA: one-way ANOVA, F(2) = 0.27, p = 0.77; caudal VTA: one-way ANOVA, F(2) = 1.27, p = 0.31; Fig. 5A,B). Dopaminergic neurons that did not project to M1 were likewise unaffected (FB^−^TH^+^c-fos^+^; rostral VTA: one-way ANOVA, F(2) = 1.03, p = 0.38, caudal VTA: one-way ANOVA, F(2) = 0.02, p = 0.98, Fig. 5C). However, the lateral distribution of FB^−^TH^+^c-fos^+^ neurons in unsuccessful learners resembled that found in animals that did learn to reach successfully (successful acquisition experiment), rather than the mostly medial expression pattern found in cage control rats that received no training.

### Effects of motor skill learning on non-dopaminergic VTA neurons

**Figure 6:**
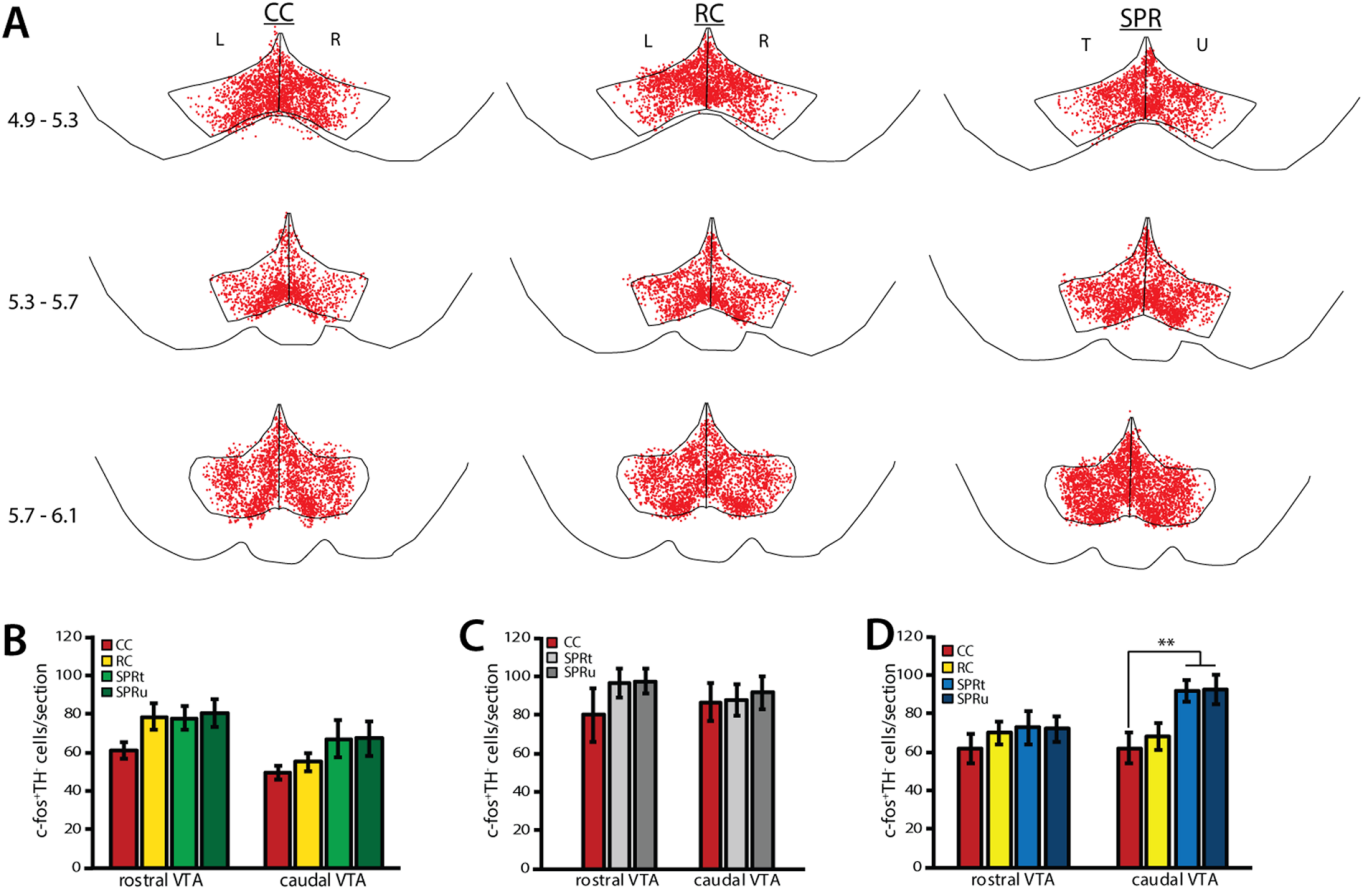
c-fos expression in non-dopaminergic (TH^−^) VTA neurons. SPR = single pellet reaching, RC = reward control, CC = cage control. T = trained, U = untrained, L = left, R = right hemisphere. Values are mean ± s.e.m. (**A**) Superposition of TH^−^c-fos^+^ (red) neurons in VTA derived from 8 animals at plateau (2 sections per position per animal). Numbers indicate position caudal of bregma. (**B**) Numbers of TH^−^c-fos^+^ neurons in rostral and caudal VTA in SA rats (N = 8). (**C**) Numbers of TH^−^c-fos^+^ neurons in rostral and caudal VTA in UA rats (N = 6). (**D**) Numbers of TH^−^c-fos^+^ neurons in rostral and caudal VTA in PL rats. (**p < 0.01 in Tukey’s HSD test)

C-fos expression was detected in non-dopaminergic (TH^−^) VTA neurons in successful learners (Fig. S1), unsuccessful learners (Fig. S2), and at plateau (Fig. 6A), as well as in reward control and cage control animals in all three experiments.

In contrast to dopaminergic (TH^+^) neurons, the number of non-dopaminergic c-fos^+^ VTA neurons (FB^−^TH^−^c-fos^+^) remained unchanged by successful motor learning or reward consumption (rostral VTA: one-way ANOVA, F(3) = 2.25, p = 0.09, caudal VTA: one-way ANOVA, F(3) = 2.03, p = 0.11; Fig. 6B). Similarly, the number of non-dopaminergic c-fos^+^ VTA neurons in unsuccessful learners was not significantly different from untrained cage controls (FB^−^TH^−^c-fos^+^; rostral VTA: one-way ANOVA, F(2) = 1.03, p = 0.38, caudal VTA: one-way ANOVA, F(2) = 0.08, p = 0.92; Figs 6D and S2). Thus, task acquisition and reward consumption during early training specifically affected dopaminergic VTA neurons.

By contrast, task execution at plateau resulted in increased numbers of non-dopaminergic neurons in caudal VTA in reaching rats (FB^−^TH^−^c-fos^+^; one-way ANOVA, F(3) = 5.58, p = 0.004), but not in rostral VTA (one-way ANOVA, F(3) = 0.56, p = 0.64; Fig. 6C) when compared to cage controls and reward controls. This increase was similar in the trained and untrained hemisphere and did not seem to be specific to subregions within the caudal VTA (Fig. 6A). Although increased caudal c-fos expression in non-dopaminergic neurons was specific to rats that learned to reach for food, the number of caudal FB^−^TH^−^c-fos^+^ neurons was not correlated to reaching success on training day 7 (trained hemisphere: Pearson’s R = −0.14, p = 0.74; untrained hemisphere: Pearson’s R = −0.35, p = 0.39).

## Discussion

Plasticity in the VTA has long been known to play an important role in the acquisition of healthy learned behaviours as well as the development of addiction by regulating motivation and reward^1,2^. Although c-fos expression in VTA neurons is low at baseline, increased c-fos expression VTA neurons has been described in a variety of learning tasks, including classical conditioning, simple operant conditioning tasks, as well as conditioning to drugs of abuse^20–24^. However, possible differential responses in dopaminergic and non-dopaminergic neuronal populations, as well as regional differences, were generally not examined in these studies.

Here we show that the dopaminergic VTA-M1 projection neurons were specifically activated in the trained hemisphere during successful acquisition of a unilateral motor skill. These projection neurons were not activated during task execution at plateau, nor by reward presentation alone. Moreover, the dopaminergic VTA-M1 projection in the untrained hemisphere showed to learning-related activation. Specific activation of the dopaminergic M1 projection during task acquisition occurred in caudal, but not rostral VTA (Table 1). Activation was not correlated with motor activity or reaching success rate. These results are in line with previous results showing that dopamine release in M1 is necessary for motor skill acquisition, but not execution^7,8^. Moreover, the lack of change in activity of the VTA-M1 projection at plateau explains why ablation of midbrain dopamine neurons^7^ or cortical application of dopamine antagonists^8^ have no effect on motor task execution at plateau. Together, our results support the role of VTA dopamine in M1 as a permissive factor for motor skill acquisition and cortical plasticity that maintains a learning state. However, successful learners only show activation of a portion of the dopaminergic VTA-M1 projection during motor skill acquisition. VTA efferents, including those to M1, are highly target specific and have few collaterals to other brain areas^9,10^. As such, forelimb and hindlimb representations in M1 are innervated by separate populations of VTA neurons^11^. The VTA-M1 projection neurons that were not activated in successful learners may thus be concerned with body parts other than the forelimb.

**Table 1:** Summary of immunofluorescence results.

cell population	training phase	rostral VTA			caudal VTA		
		SPRt	SPRu	RC	SPRt	SPRu	RC
FB^+^TH^+^c-fos^+^	SA	—	—	—	↑	—	—
	UA	—	—	—	—	—	—
	PL	—	—	—	—	—	—
FB^−^TH^+^c-fos^+^	SA	↑	↑	↑	↑*	↑*	↑
	UA	—	—	—	—	—	—
	PL	↑	↑	↑	↑	↑	↑
FB^−^TH^−^c-fos^+^	SA	—	—	—	—	—	—
	UA	—	—	—	—	—	—
	PL	—	—	—	—	—	—

Arrows indicate increased numbers of cells compared to cage control animals. Asterisks indicate significantly higher numbers in SPR rats than in reward controls. SA = successful acquisition; UA = unsuccessful acquisition; PL = plateau performance; SPRt = single pellet reaching, trained hemisphere; SPRt = SPR, untrained hemisphere; RC = reward control.

In contrast to successful learners, rats that failed to successfully acquire the skilled reaching task did not show increased activation of dopaminergic VTA neurons compared to untrained, non-rewarded controls. However, the distribution of TH^+^cfos^+^ neurons in unsuccessful learners resembles that of successful learners, suggesting that some dopaminergic VTA neurons may be involved in task execution, irrespective of reaching success. This is in line with a role for VTA dopamine release in sustaining and organizing ongoing task-related behaviors^25–27^.

Rostral and caudal VTA are known to be functionally distinct. This is in part due to differences in projections to and from these subregions^16,28–30^, but also to differential responses to stimuli in distinct neuronal populations^30–32^. In general, rostral and caudal VTA show opposing responses to stimuli, and stimulation results in opposing behavioural effects^16,17^. For example, overexpression of CREB or phospholipase C γ1 (PLCγ1) in rostral VTA increased rewarding effects of drugs, whereas overexpression in caudal VTA resulted in an aversive response^13,14,33^. Knockdown of CREB in these regions had the opposite effect^14^. Similarly, AMPA-receptor subunit GluR1 overexpression in rostral VTA enhances the rewarding effects of morphine, but causes aversive reactions in the caudal VTA^34^. Conversely, administration of morphine and cocaine caused more induction of c-fos and CREB in caudal than in rostral dopaminergic VTA neurons^14,20^. Thus, caudal VTA plasticity is involved in both addictive and aversive responses to drugs^12,13,15,18,19^, whereas activation of rostral VTA augments their rewarding effects^35,36^. The roles of VTA subregions in mediating natural rewards and behaviours have been less well studied. Overexpression of PLCγ1 in rostral VTA increased, and in caudal VTA decreased sucrose consumption, indicating that their distinct roles generalize at least partly to natural rewards^13^. Our results suggest a functional segregation of the VTA in mediating the behavioural components of skilled reaching during different phases of learning as well, although future experiments will be required to elucidate the precise mechanisms by which the described activity patterns drive behaviour. We hypothesize that c-fos expression in dopaminergic rostral VTA neurons may have been related to food reward consumption during skill acquisition, as expression patterns are similar in reaching rats and untrained reward controls. By contrast, activation of caudal VTA may be induced either by a different, non-reward signal occurring during motor skill acquisition or by the combination of reward and skill acquisition, but not by reward consumption alone. This hypothesis requires testing in future experiments. At plateau, the functional separation of rostral and caudal dopaminergic VTA neurons was no longer present and c-fos expression in both rostral and caudal dopaminergic VTA neurons was related to reward consumption.

Activation of caudal VTA neurons during successful motor skill learning seems inconsistent with their role in regulating responses to aversive stimuli such as stress and pain^13^, although a mild stressor did not induce VTA c-fos expression^37^. Learning to reach for pellets may be considered mildly stressful because access to food pellets is limited and obtaining them requires effort. However, all rats that were trained to perform the skilled reaching task, including unsuccessful learners, continued reaching despite limited initial success. This suggests that this task is not stressful or aversive enough to avoid. Additionally, unsuccessful learners do not show increased numbers of dopaminergic or non-dopaminergic c-fos^+^ VTA neurons, even though they receive very little reinforcement. Rather than reflecting aversive stimuli, activation of caudal VTA non-dopaminergic neurons in successful learners at plateau may be related to routine forelimb activity or habit formation^38^. Another possibility is that activation of caudal VTA neurons during task acquisition and at plateau is caused by nonspecific motor activity (e.g. running in the training cage). While wheel running can induce rhythmic firing in non-dopaminergic VTA neurons^39^, c-fos expression is only induced in these neurons after long-term running and not after a short period of activity^40^. The amount of locomotor activity during a 30-minute SPR session is much lower than that of a typical wheel running session and therefore unlikely to result in increased c-fos expression. Moreover, overall motor activity at plateau was similar to that observed during successful task acquisition, but rostro-caudal c-fos expression patterns were different.

Reward control rats showed relatively high VTA c-fos expression in both the acquisition and the plateau experiment. This may be a consequence of conditioned responses of these rats to the delivery of food rewards, as well as to the sound of the reaching rat’s cage door opening. Although food reward delivery is controlled by the outcome of a reaching attempt and not by any behavior of rats in the reward control group, they reliably investigate the food tray after hearing the door opening. On the final training day, when reaching rats achieve relatively high success rates, this sound is often followed by pellet delivery, rewarding the conditioned approach behavior of the reward control group. VTA c-fos expression in these animals may therefore be caused by a combination of pellet consumption^21,22^, being placed in a reward-paired environment^21,22,41,42^, and the conditioned approach behavior^21^.

Repeated exposure to rewards, resulting in habituation, typically no longer induces VTA c-fos expression^43^. However, reaching rats and reward controls in the plateau experiment had higher numbers of c-fos^+^ neurons than untrained, unrewarded controls. Rats in the plateau experiment have been carrying out the reaching task with maximum success for approximately 3 days prior to sacrifice. Even though the number of consumed pellets remains stable during these days, the 3-day period may not be sufficient to result in complete habituation to stable rewards and thus still results in elevated numbers of c-fos^+^ nuclei. In reaching rats, continued c-fos expression is possibly related to movement refinement that occurs for a number of days after success rates have plateaud^44^.

The use of c-fos expression to assess neuronal activation is a limitation of this study. While VTA c-fos has been shown to be involved in learning, some VTA neurons may recruit other transcription factors when activated^14,45^. Additionally, c-fos has limited temporal sensitivity as a marker of neuronal activity and cannot be used to distinguish between tonic and phasic firing patterns of dopaminergic VTA neurons. Instead, VTA c-fos expression is likely the result of cumulative neuronal activity during a training session^43,46^. A causal relationship between motor skill acquisition and neuronal activity in different cell types and VTA subregions, as well as the temporal activity patterns of these neurons, will need to be investigated using other methods.

In conclusion, motor skill learning is accompanied by region- and cell-type specific activity patterns in the VTA. These activity patterns are partly specific to motor skill acquisition and may support cortical plasticity involved in motor skill acquisition and consolidation. Our results are in line with a shift of dopaminergic neuronal activity in response to reward-predictive cues during learning (e.g. successful grasping of the pellet), to activity that reflects reward consumption at plateau and no longer differs between reaching rats and non-reaching controls. Surprisingly, non-dopaminergic caudal VTA neurons seem to play a role in task execution at plateau, as shown by increased c-fos expression in caudal VTA at plateau. Thus, the shift from cue-evoked VTA responses to reward-related responses seems to involve differential activation of VTA subregions as well as cell types. The precise behavioural signal that induces motor-learning specific VTA activation remains to be elucidated.

## Methods

### Animals

60 male Long-Evans rats (10–12 weeks old, Janvier Labs) were used. Rats were group-housed in standard IVC cages (T1500) with a 12-hour light, 12-hour dark cycle. All experimental procedures were carried out during the first 4 hours of the dark period. Animals were kept on a feeding schedule in which they were fed 50 g/kg of chow daily after training throughout the entire experiment. This feeding schedule did not cause any significant weight loss. Water was available *ad libitum*. All procedures were approved by the Animal Experimentation Committee of the Canton of Zurich (license ZH50/2012) and were carried out in accordance with national and institutional guidelines and regulations.

### Experimental design

To distinguish effects of motor learning from reward processing in the VTA during motor skill learning, animals were assigned to one of 3 experimental groups that were tested simultaneously. Rats in the skilled reaching (SPR) group were trained to perform a single pellet reaching task. A reward control (RC) rat was trained in parallel and received a food pellet whenever the SPR rat successfully obtained one (Fig. 1A). Finally, a cage control (CC) rat was kept in a training cage during the training period of matched SPR and RC animals, but received neither pellets nor training. To study differences in VTA activation at different learning stages, the rats were trained for either 3 (task acquisition) or 7 (task execution at plateau) consecutive days and were sacrificed 1 hour after the final reaching attempt of the last training session (Fig. 1B). SPR rats that successfully learned to reach for food within three reaching sessions were included in the successful acquisition experiment with their matched controls (SA, n = 8 per group). 6 SPR rats demonstrated poor learning ( < 10% success in session 3) and were included in the unsuccessful acquisition experiment (UA, N = 6 per group). 24 additional rats were trained for 7 consecutive days in the plateau experiment (PL, N = 8 per group). Learning- or reward-related activation of dopaminergic VTA-neurons projecting to M1, identified by retrograde tracing, was assessed by immunofluorescence for tyrosine hydroxylase (TH) and c-fos.

### Behavioural training

A 15 × 40 × 30 cm (W × L × H) cage with a vertical window (1 cm wide, lower edge 5 cm above the cage floor) in the front wall and a sensor in the back wall was used for all experimental groups. Skilled reach training for the SPR group was performed as described previously^47^. In brief, after 2 days of habituation to the cage, SPR animals were pre-trained to open the motorized door covering the cage window by touching the sensor in the back of the cage. Opening the window allowed access to a single food pellet that could be reached by tongue (45 mg dustless precision pellets, Bio-Serv). Each pre-training session consisted of 100 trials (opening the window and retrieving a pellet) or 60 minutes, whichever came first. After 4 pre-training days, when the animal could perform 100 trials within 20 minutes, the retrograde tracer FastBlue (FB, Polysciences) was injected in both hemispheres. Animals recovered for at least 36 hours after surgery, followed by three more pre-training sessions for SA and UA experiments and one pre-training session for the PL experiment to allow sufficient time for the FastBlue to reach the soma at the end of the experiment.

Subsequent skilled reaching training was similar to pre-training, but the pellet was now placed on a pedestal located 1.5 cm outside of the cage where it could only be retrieved by using a forepaw. A reaching trial was scored as either successful (pellet is retrieved from the pedestal and eaten) or failed (pellet is pushed off the pedestal or dropped during paw retraction). Reaching sessions consisted of 100 trials each. The final reaching session (session 3 and 7 in the acquisition and plateau groups, respectively) was optimized for c-fos expression and consisted of 200 trials, assuring a training period of at least 30 minutes. Trials were recorded on video for later verification and additional analyses. Each SPR rat was trained in parallel to one reward control (RC) and one cage control (CC) rat. RC animals received a food pellet in a small tray in the training cage whenever the matched SPR animal successfully obtained a pellet. The CC rat did not receive any pellets, but was otherwise exposed to an identical training cage environment. Of the trained rats, 10 showed a preference for the left and 12 for the right forepaw.

Rats were excluded from analysis if retrograde tracing was unsuccessful in either hemisphere, if FB injection had caused excessive damage to M1 (microscopic analysis revealed more damage than the expected needle tracks), or if they did not attempt to reach out at all.

Animals were excluded from successful acquisition (SA) and plateau (PL) experiments if they showed no improvement in reaching performance during the first 2 training sessions and did not achieve a minimum success rate of 10%, or if they did not attempt to reach out at all. Poorly performing rats in the SA experiment were trained for a third and final session and were included in the unsuccessful acquisition (UA) experiment. As these UA rats consumed almost no reward pellets, RC rats were not analysed for these experiments.

### Retrograde tracing

Animals were anaesthetized using ketamine (100 mg/kg, i.p., Gräub) and xylazine (10 mg/kg, i.p., Streuli). Additional ketamine injections (30 mg/kg, i.p.) were given as needed. Body temperature was kept stable at 37 °C using a heating blanket (Kent Scientific). The head was fixed in a stereotaxic frame (David Kopf Instruments) and two small craniotomies were made over M1 (1–3 mm lateral, 1–3 mm rostral of bregma). Neurons projecting to M1 were labelled with the fluorescent retrograde tracer FastBlue (FB, Polysciences). Three deposits of 250 nl FB suspension (1% FB, 1% DMSO in PBS) were made covering the forelimb representation in M1 in each hemisphere (Depth: 900 µm. Positions relative to bregma: 2 mm rostral, 2 mm lateral; 2.5 mm rostral, 2 mm lateral; 2.5 mm rostral, 2.5 mm lateral). FB suspension was injected over a 60-second period using a microliter injection pump (Quintessential Stereotaxic Injector, Stoelting) and microsyringe with a 26 G needle (Hamilton). The needle was left in place for 5 minutes after injection before it was slowly retracted. After all injections were completed, the craniotomy was covered with dental cement (Venus Flow, Heraeus) and the scalp was closed. Animals received injections of caprofen (Rimadyl, 5 mg/kg, s.c., Pfizer) and enrofloxacin (Baytril, 5 mg/kg, s.c., Bayer) shortly before recovering from anaesthesia. Rats were allowed to recover for at least 36 hours before pre-training was resumed.

### Tissue processing and immunofluorescence

Unless stated otherwise, all chemicals were obtained from Sigma Aldrich. One hour after the final reaching attempt, rats were perfused transcardially with 0.1 M phosphate-buffered saline (PBS) followed by 4% paraformaldehyde (w/v, PFA) in PBS. Brains were post-fixed in 4% PFA in PBS for 24 hours, cryoprotected in 30% sucrose in PBS, and frozen.

FastBlue injection placement and dye spread was assessed based on 50 µm thick coronal forebrain sections. These sections were counterstained with Nuclear Yellow (Hoechst S769121, Molecular Probes) and mounted using VectaShield mounting medium (Vector Labs).

50 µm thick coronal brain sections containing VTA were cut on a cryostat (Leica). The left hemisphere of each section was marked by a needle puncture. Every fifth section was stained and analysed. Free-floating VTA sections were washed in Tris-buffered saline (TBS), incubated in TBS^+^ (2% bovine serum albumin and 0.3% Triton-X100 in TBS) for 45 minutes, after which they were incubated with primary antibodies against tyrosine hydroxylase (1:500, monoclonal mouse-anti-TH MAB318, Millipore) and c-fos (1:500, monoclonal rabbit-anti-c-fos 9F6, Cell Signalling Technology) in TBS + for 48 hours. Sections were then washed in TBS and incubated in TBS^+^. Staining was visualized by incubation with Cy3- and FITC- conjugated secondary antibodies (goat-anti-rabbit IgG-Cy3 (1:500), and goat-anti-mouse IgG-FITC (1:500), Jackson Immunoresearch) in TBS^+^ for 3 hours. Sections were mounted using Prolong Gold mounting medium (Molecular Probes). Sections in which the primary antibodies were omitted were included as negative controls for the staining procedure. No immunopositive cells were detected in these sections.

### Tissue and behavioural analysis

Reaching task performance was calculated as the percentage of successful reaching attempts out of the total number of possible attempts for each session. Additionally, the number of grabbing attempts was counted for each trial and average number of grabbing attempts per trial was calculated. A grabbing attempt was defined as extension of the paw from the cage window followed by complete or partial retraction.

The average number of grabbing attempts per trial could not be calculated for one SPR animal in the UA experiment because the experimenter partially blocked the view of paw and pedestal on the video footage. The number of grabbing attempts in successful trials could not be calculated for 3 unsuccessful learners, because these animals did not achieve any successful trials. The distance walked in a training session was estimated based on the number of times each rat walked the length of the training cage. VTA sections were analysed using unbiased stereology using a Zeiss Imager Z1 fluorescence microscope (Zeiss) and StereoInvestigator 8 (MBF Bioscience). Left and right VTA were outlined based on TH immunoreactivity in sections 5.4 to 7.5 mm caudal of bregma. Triple labelled (FB^+^ TH^+^c-fos^+^), double labelled (TH^+^c-fos^+^, FB^+^c-fos^+^, FB^+^TH^+^) and single labelled (c-fos^+^) neurons were counted at 40x magnification in the optical fractionator workflow using a 150 × 150 µm counting frame and an optical dissector height of 22 µm on a 150 × 150 µm grid, ensuring complete coverage of the VTA. Statistical analyses were performed using SPSS 23 (IBM).

Sections including FastBlue injection sites were photographed at 5x magnification with the same microscope using StereoInvestigator. Needle placement, cortical damage and dye spread were assessed and outlined in ImageJ (NIH). Illustrative micrographs of single, double and triple labelled VTA neurons were taken using StereoInvestigator at 10x and 40x magnification and were processed to adjust contrast and background in ImageJ. Changes in reaching performance in the SPR group were analysed using repeated measures ANOVA. Cell counts and motor activity were compared between groups using one-way ANOVA with post-hoc Tukey’s HSD tests where appropriate. All values are reported as mean ± s.e.m, unless stated otherwise. The datasets generated during the current study are available from the corresponding author on request.

## Electronic supplementary material

Supplementary figures
