# Supplementary material for: Motor skill learning and reward consumption differentially affect VTA activation
Source: Sci Rep. 2018 Jan 12;8:687. doi: 10.1038/s41598-017-18716-w (PMC5766527; doi:10.1038/s41598-017-18716-w)
Supplement: Supplementary file 1 — Supplementary figures [file 41598_2017_18716_MOESM1_ESM.pdf]

Title:

Motor skill learning and reward consumption differentially affect VTA activation

Authors:

Susan Leemburg[1]
Tara Canonica[1,3]
Andreas Luft[1,2*]

[1] Division of Vascular Neurology and Rehabilitation, Department of Neurology, University Hospital Zurich, Zurich, Switzerland
[2] cereneo Center for Neurology and Rehabilitation, Vitznau, Switzerland
[3] College of Biomedical and Life Sciences, School of Psychology, Cardiff University, Cardiff, United Kingdom
*corresponding author

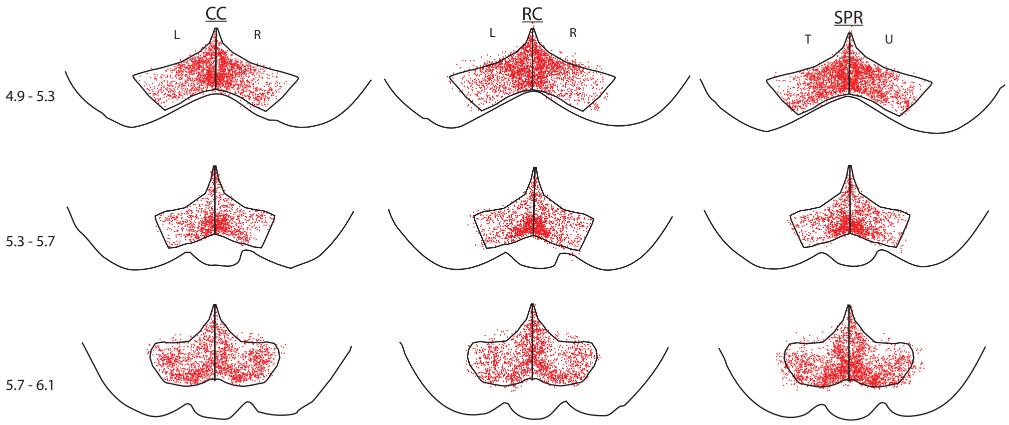

Fig. S1 Superposition of c-fos^{+}TH^{-} neurons in VTA during successful acquisition (N=8 animals per group, 2 sections per animal). SPR= skilled reaching, RC= reward control, CC= cage control. T= trained, U= untrained, L= left, R= right hemisphere. Numbers indicate position caudal of bregma.

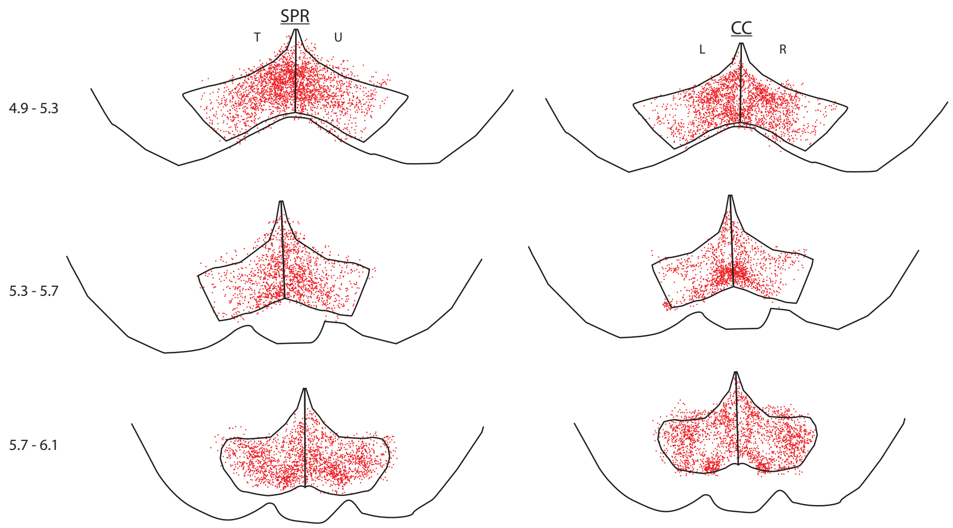

Fig. S2 Superposition of c-fos$^+$TH$^-$ neurons in VTA during unsuccessful acquisition (N=6 animals per group, 2 sections per animal). SPR= skilled reaching, RC= reward control, CC= cage control. T= trained, U= untrained, L= left, R= right hemisphere. Numbers indicate position caudal of bregma.
